# Physical soil architectural traits are functionally linked to carbon decomposition and bacterial diversity

**DOI:** 10.1038/srep33012

**Published:** 2016-09-12

**Authors:** S. M. F. Rabbi, H. Daniel, P. V. Lockwood, C. Macdonald, L. Pereg, M. Tighe, B. R. Wilson, I. M. Young

**Affiliations:** 1 School of Life and Environmental Sciences, University of Sydney, Sydney, NSW, Australia; 2 Plant, Soil and Environment Systems, School of Environmental and Rural Science, University of New England, Armidale, NSW, Australia; 3 Hawkesbury Institute for the Environment, Western Sydney University, Sydney, Australia; 4 School of Science and Technology, University of New England, Armidale, NSW, Australia

## Abstract

Aggregates play a key role in protecting soil organic carbon (SOC) from microbial decomposition. The objectives of this study were to investigate the influence of pore geometry on the organic carbon decomposition rate and bacterial diversity in both macro- (250–2000 μm) and micro-aggregates (53–250 μm) using field samples. Four sites of contrasting land use on Alfisols (i.e. native pasture, crop/pasture rotation, woodland) were investigated. 3D Pore geometry of the micro-aggregates and macro-aggregates were examined by X-ray computed tomography (μCT). The occluded particulate organic carbon (oPOC) of aggregates was measured by size and density fractionation methods. Micro-aggregates had 54% less μCT observed porosity but 64% more oPOC compared with macro-aggregates. In addition, the pore connectivity in micro-aggregates was lower than macro-aggregates. Despite both lower μCT observed porosity and pore connectivity in micro-aggregates, the organic carbon decomposition rate constant (Ksoc) was similar in both aggregate size ranges. Structural equation modelling showed a strong positive relationship of the concentration of oPOC with bacterial diversity in aggregates. We use these findings to propose a conceptual model that illustrates the dynamic links between substrate, bacterial diversity, and pore geometry that suggests a structural explanation for differences in bacterial diversity across aggregate sizes.

Soil physical architecture (e.g. porosity, pore size distribution, pore connectivity) controls soil microbial activity over a range of spatial scales^1^. At spatial scales relevant to biological processes (i.e. carbon decomposition) and soil management (i.e. tillage, land use change), pore geometry controls oxygen diffusion rate, water flow and nutrient supply for microbial communities and vascular plants^2^. Pore architecture also controls microbial community structure by regulating these factors and most importantly, by impacting on the competition and predation among microbial groups^3^. Any input of organic carbon in soil is subject to microbial decomposition and the rate of this decomposition depends on soil texture, moisture content, temperature and nutrient availability in the soil. Soil micro-aggregates may form around decomposing organic carbon and thus organic carbon becomes encapsulated inside these aggregates. The physical protection of organic carbon inside the soil matrix is thought to slow down its decomposition rate primarily due to lack of microbial penetration into micro-aggregates^4^,^5^. Moreover, the oxygen diffusion rate into the micro-aggregates is also low compared to bulk soil as a consequence of the change in pore geometry at the early stage of aggregate formation^6^,^7^. Such changes in pore geometry, and the physical protection mentioned above, may allow aggregates to store organic carbon over a long period of time. The mean residence time of organic carbon in aggregates may vary from 30 to 500 years depending on climatic and edaphic factors^8^.

Microbial community structure can vary spatially even at the micrometer scale^9^,^10^,^11^,^12^. The microbial community structure and function is highly dependent on soil architecture, the nature and properties of organic carbon, and the plant species present^13^,^14^,^15^,^16^,^17^,^18^. Recently, Ruamps *et al*.^1^ concluded that the function of microbes in soil was largely controlled by pore geometry of soil. This is well corroborated with earlier works by other authors^3^,^11^,^19^. Most of these works were based on analysis of 2D pore geometry and biogeography of microbes in soil. However, analyzing pore architecture in 3D is essential to understand the relationship between pore geometry and microbial habitat and functions^20^,^21^.

The examination of undisturbed soil aggregates by X-ray computed micro-tomography (μCT) allows non-destructive quantification of 3D pore geometry, which in turn allows direct links to be made between the 3D pore geometry and microbial processes in soil. Several works using μCT have demonstrated the effect of organic carbon addition on pore geometry of soil aggregates^22^,^23^,^24^. However, none of the previous studies were able to relate the 3D pore geometry to the functions of soil^25^. Moreover, there appears to be a feedback loop between organic carbon concentration and pore geometry of soil^26^. Both organic matter concentration and pore geometry regulates the carbon decomposition rate and microbial diversity in soil, but it is still unclear whether the influence of pore geometry on carbon decomposition and microbial diversity is stronger compared with carbon concentration in soil. The objective of the present study was to investigate the influence of pore geometry on organic carbon decomposition rate and bacterial diversity in soil aggregates. We also sought to analyze functional relationships among pore geometry, organic carbon decomposition and bacterial diversity in soil aggregates using structural equation modelling (SEM).

## Results

### Pore geometry of aggregates

**Figure 1:**
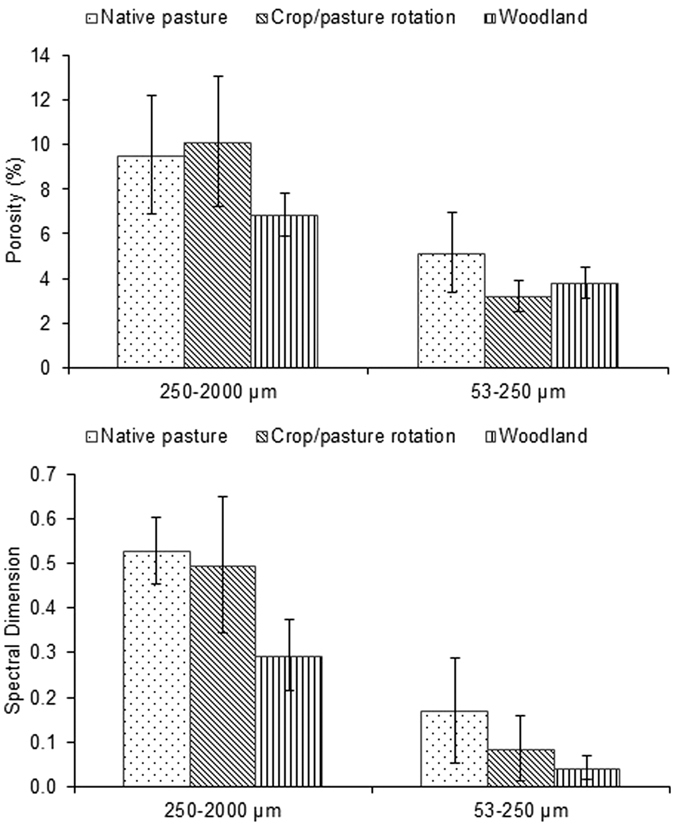
Porosity and spectral dimension of macro-aggregates (250–2000 μm) and micro- (53–250 μm) under the sampled land uses.

**Figure 2:**
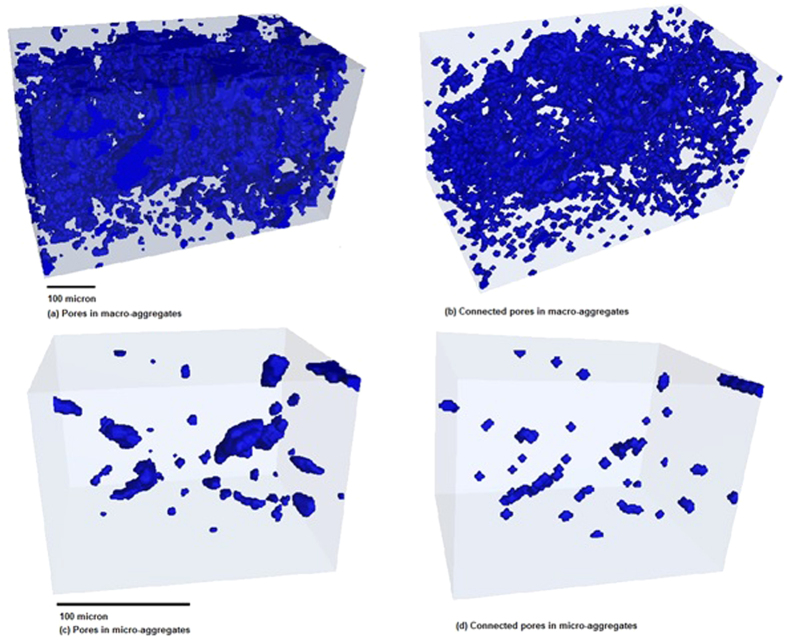
Example of 3D images of a macro-aggregate (250–2000 μm) and micro-aggregate (53–250 μm) showing pores and connected pores in dark blue and soil matrix in light blue. The connected pore networks were the skeletonized pore spaces of macro- and micro-aggregates.

**Figure 3:**
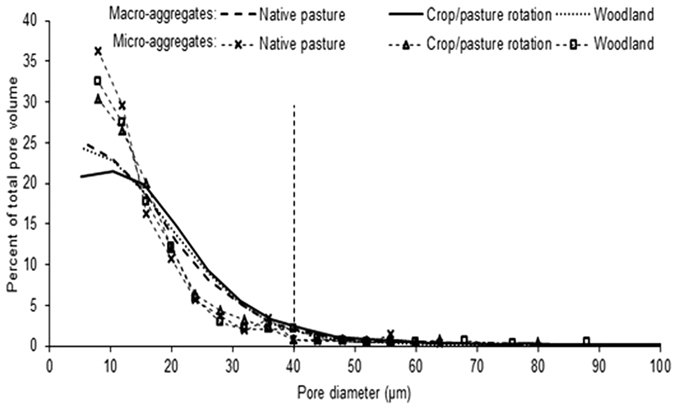
Pore size distribution of macro-aggregates (250–2000 μm) and micro-aggregates (53–250 μm) under contrasting land uses at different sites. The vertical broken line on x-axis represents the cut-off below which pore size distribution of micro-aggregates differs from macro-aggregates.

Pore geometry of soil aggregates under three different land uses (i.e. native pasture, crop/pasture rotation and woodland), collected from 4 sites, was evaluated using μCT. The average μCT observed porosity (henceforth porosity) of macro-aggregates was 54.4% greater than that of micro-aggregates (p < 0.05) (Figs 1 and 2). The spectral dimension (i.e. a mathematical expression of pore connectivity) of macro-aggregates was significantly (p < 0.05) higher compared to micro-aggregates, indicating that the macro-aggregates had more connected pores than the micro-aggregates. The percent volume of pores occupied by <40 μm pores was higher than >40 μm pores in both macro- and micro-aggregates. Macro-aggregates had 27% more 20–40 μm pores compared to micro-aggregates (p < 0.05). However, <20 μm pores were significantly higher in micro-aggregates than macro-aggregates (p < 0.05) (Fig. 3). The pores >40 μm occupied only a small volume (<4%) of micro-aggregate porosity but up to 11% of macro-aggregate porosity. The effect of land uses on aggregate porosity, spectral dimension and pore size distribution was not statistically significant.

### oPOC and Ksoc in soil aggregates

**Figure 4:**
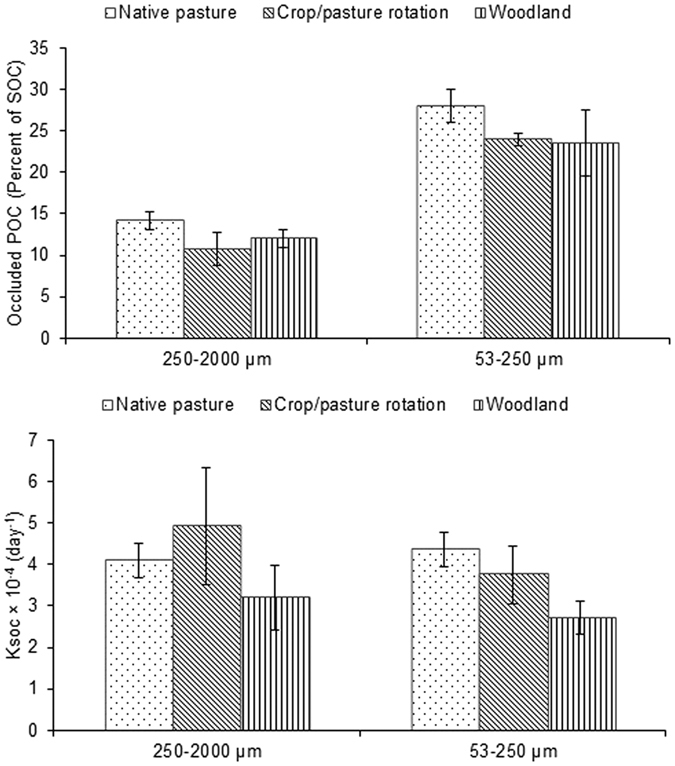
Occluded particulate organic carbon (oPOC) (as percent of total organic carbon) and Organic carbon decomposition rate constant (Ksoc) of macro- and micro-aggregates under contrasting land uses at different sites.

Occluded POC concentration and Ksoc of aggregates under native pasture, crop/pasture rotation and woodland collected from 4 different sites were measured. On average the micro-aggregates had 64% higher oPOC than macro-aggregates (p < 0.05) (Fig. 4). Despite the higher occluded carbon percentage in the micro-aggregates, Ksoc was statistically similar between micro- and macro-aggregates (Fig. 4). The effects of land uses on oPOC and Ksoc were not statistically significant.

### Diversity of bacteria in aggregates

**Figure 5:**
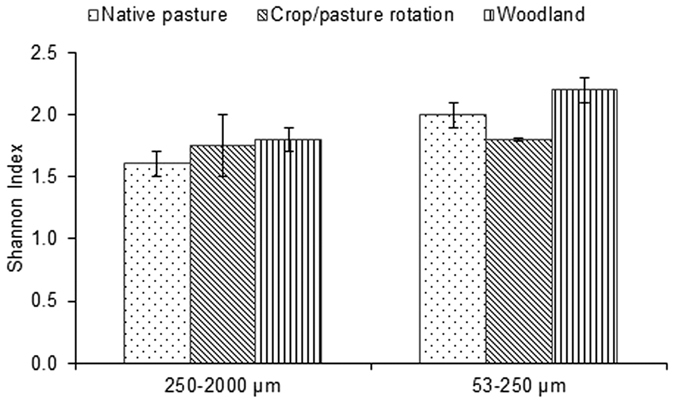
Diversity of bacteria (Shannon Index) in micro- and macro-aggregates under contrasting land uses.

The diversity of bacteria in aggregates under three different land uses (i.e. native pasture, crop/pasture rotation, woodland) of one sampling site was measured. The micro-aggregates had significantly higher bacterial diversity than macro-aggregates (p < 0.05). Although macro- and micro-aggregates under woodland appeared to have higher diversity compared to under native pasture and crop/pasture rotation, differences in bacterial diversity under different land uses were not statistically significant (Fig. 5).

### Relationship between pore geometry, oPOC, Ksoc and bacterial diversity

**Figure 6:**
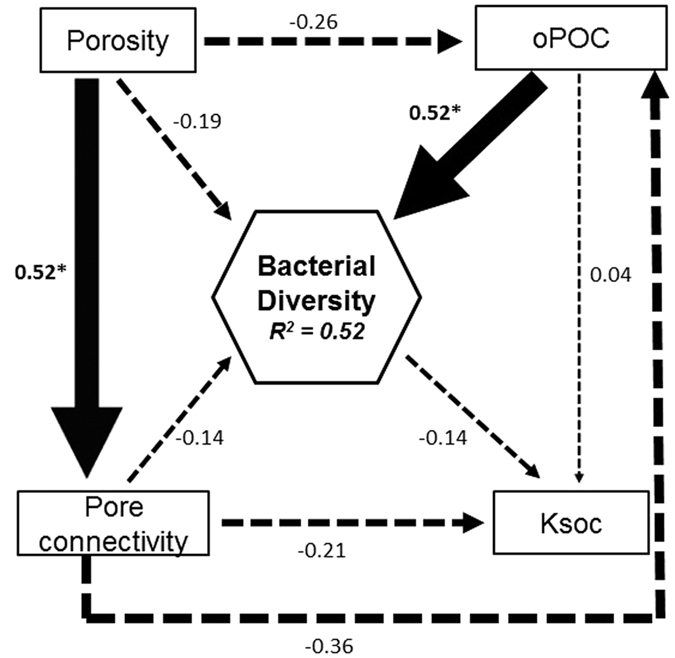
Relationship of pore geometry with carbon decomposition rate occluded POC (oPOC) and bacterial diversity. The model attained an acceptable fit (χ^2^ = 0.342, p = 0.559, df = 1, Bootstrap p = 0.667, RMSEA = 0.0 p = 0.565, AIC = 38). The numbers adjacent to the arrows represent standardized path coefficients, analogous to regression weights. The width of each arrow is indicative of effect size. Continuous arrows indicate significant (p < 0.01) positive or negative relationships, whereas dashed arrows indicate non-significant relationships (p > 0.05). The proportion of variance of bacterial community structure explained (R^2^) is shown in the box for bacterial diversity.

The structural equation modelling (SEM) was performed using a subset of our data to evaluate the relationship of bacterial diversity with oPOC, Ksoc and pore geometry of aggregates. The SEM analysis showed that porosity, pore connectivity, Ksoc and oPOC could explain 52% of the total variation in bacterial diversity in aggregates (Fig. 6). The standardized path coefficient indicated that oPOC had a significant positive correlation (r = 0.52, p < 0.05) with bacterial diversity in aggregates. The pore connectivity was negatively correlated with porosity (r = 0.52, p < 0.05). Porosity and pore connectivity had no significant correlations with oPOC or Ksoc.

## Discussion

The physical architecture of soil (how porous and connected it is, and how such pores are distributed in 3D) controls the microbial functions of soil. In turn, the microbes (through processes such as degradation of organic matter and secretion of polymers) have large and significant impacts on soil architecture at antecedent soil moisture^3^. The novel approach of the current work integrates results of μCT, SOC mineralization, and molecular analyses to understand the functional relationship between pore geometry and bacterial diversity in soil aggregates using structural equation modelling.

We observed high bacterial diversity in micro-aggregates compared with macro-aggregates, which was associated with a significant positive relationship between diversity and oPOC in aggregate. Previous research has demonstrated strong positive relationships between organic carbon concentration and microbial community structure^27^, but this work is a novel demonstration of this link with 3D structural data.

The spatial biogeography of soil microbes is diverse. The microbial community varies non-randomly from large scale (~1 m) to micro-scale (i.e. aggregate scale)^28^,^29^. The function of microbial communities of soil is dependent on pore geometry and available substrate concentration^30^, which makes the consideration of the 3D pore architecture critical in determining such relationships. The pore geometry, especially pore connectivity, is often considered as the route for microbial interaction with other microbes and available substrates^1^,^31^. Clusters of active microorganisms are often reported to be found near preferential flow paths of water and nutrients^3^,^11^. The evidence from our work suggests that despite high available substrate (i.e. oPOC) and bacterial diversity, Ksoc of micro-aggregates was similar to that of macro-aggregates, which is an indication that the environment within micro-aggregates might be less favorable for decomposition compared with macro-aggregates. However, since we used a small dataset in the SEM to evaluate the relationship of Ksoc with pore geometry, we took the non-significant negative association between Ksoc and pore connectivity to imply a potential indication that pore connectivity played a regulatory role in organic carbon decomposition in soil aggregates. While the current work was a survey based study, and thus causal inferences drawn from the SEM need to be interpreted with caution, we based this inference partly on relevant recent work. Specifically, Bouckaert *et al*.^32^ reported that the rate of carbon decomposition in soil pores was influenced by pore neck diameter and water retention properties of soil. The porosity and pore connectivity are related and important components of the microbial habitat that have ability to regulate the transport of substrates, water and oxygen in the microbial hotspots. Pore size of about 10 μm defines the boundary between free drainage water and capillary water^33^. This implies that anoxic conditions are more likely to prevail in micro-aggregates and within a portion of macro-aggregates^34^. At water potentials near saturation and in occluded pores spatially isolated from an oxygen supply, decomposition by aerobic microbes becomes slow. This results in a low microbial growth efficiency (i.e. the amount of new biomass carbon produced per unit substrate metabolized)^35^. The negative association between Ksoc and pore connectivity found in this work further suggests micro-aggregates could have higher water retention than macro-aggregates due to the higher number of 20–40 μm size pores in the macro-aggregates. The high water retention would limit the oxygen diffusion rate in micro-aggregates, which in turn would influence the rate of decomposition.

**Figure 7:**
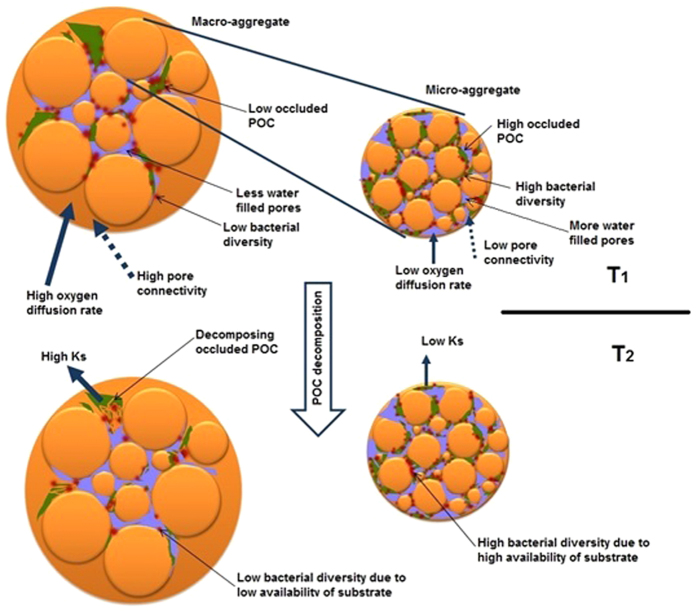
A schematic diagram showing the relationship of bacterial diversity with oPOC, pore geometry and organic carbon decomposition rate at two time points (T_1_ and T_2_). T_1_ represents an arbitrary stage of oPOC decomposition in soil aggregates. As decomposition advances from T_1_ to T_2_, compared to macro-aggregates, micro-aggregates would retain more oPOC and support diverse bacterial community due to slower decomposition rate.

Considering the positive relationship between oPOC and bacterial diversity and the negative relationship between Ksoc pore connectivity, we have developed a conceptual model that brings together both the diversity-architecture interactions and the impact of the food reserve (in this case oPOC). This is summarized in Fig. 7. We propose that when decomposition of oPOC proceeds in soil aggregates, the Ksoc will be high in macro-aggregates due to the presence of highly connected, relatively large pores, which would facilitate high rates of oxygen diffusion^36^. The high rate of decomposition reduces the substrate concentration in macro-aggregates and subsequently reduces the bacterial diversity. Moreover, it is generally expected that saprophytic fungi is more abundant in macro-aggregates than micro-aggregates^28^. This means the competition for available substrate between saprophytic fungi and bacteria might also reduce the bacterial diversity in macro-aggregates. Since the pores in micro-aggregates are smaller and less connected than macro-aggregates, the Ksoc is slower compared to macro-aggregates. Moreover, oPOC decomposition rate would be further reduced by presence of water filled pores in micro-aggregates. The slow oPOC decomposition in micro-aggregates would provide high substrate availability for a diverse bacterial community.

Thus, the bacterial diversity in micro-aggregates would be dependent on the availability of substrate for microbes but the functions (i.e. organic carbon decomposition) of bacteria would be influenced by the pore geometry of aggregates. Moreover, there is a feedback between pore geometry and soil organic carbon (i.e. the decomposition of organic carbon reorganizes the pore geometry of the soil and the modified pore geometry influences the rate of decomposition^18^,^26^). The current work using high resolution μCT images of micro- and macro-aggregates and molecular biological techniques provides evidence that at the micro-scale the availability of substrate influences bacterial diversity in soil but the pore geometry has the potential to influence bacterial function. Such micro-scale analysis does not account for preferential flow paths of nutrients that exist between field scale soil structural units, which requires further detailed investigation.

## Conclusion

Using novel μCT and SEM we investigated the influence of pore geometry on organic carbon decomposition rate in micro- and macro-aggregates. The concentration of substrate (oPOC) determined the bacterial diversity but pore geometry influenced the organic carbon decomposition rate within aggregates. These findings provide a platform for a conceptual model that proposes potential feedbacks between pore geometry, bacterial diversity, and substrate availability that can help target further investigations into the dynamic nature of soil spatial biochemistry and physical architecture. Further research should be directed towards the evaluation of bacterial diversity in aggregates using SEM analysis or other complex data analysis techniques with pore geometry, microbial gene sequencing, quantity and quality of organic carbon, decomposition rate and soil water potential data under controlled settings with a temporal aspect to the investigations.

## Materials and Methods

### Study area and soil sampling

The study was conducted in four sites in the Northern Tablelands region of NSW, Australia. Soil samples were collected from the surface (0–10 cm) of Alfisols^37^ of three contrasting land uses of (i) native pasture (ii) crop/pasture rotation and (iii) woodland. The native pasture sites were composed solely of native perennial grasses including Red Grass (*Bothriochloa macra*), Wire Grass (*Aristida ramosa*), and Wallaby Grass (*Austrodanthonia* spp.). The recently sown crops at the sites were fescue (*Festuca arundinace*), ryegrass (*Lolium perenne*), triticale (*Triticale hexaploide* ) and millet. The woodlands at all sites consisted of *Eucalyptus* spp. dominated by Blakely’s Red Gum (*E. blakelyi*) and Yellow Box (*E. melliodora*).

The detailed description of management history of the sites and characteristics of the soils can be found in Rabbi *et al*.^38^ (Supplementary Material S1). Soil samples were collected from each land use by selecting 3 separate blocks (50 m × 50 m) along the slope of each paddock. Three soil core samples were collected from each block by inserting a cylindrical metal core of 5 cm in diameter to 10 cm depth. Bulk soil samples (0–10 cm) were also collected from these 3 blocks of a paddock.

### Aggregate preparation

Rabbi *et al*.^39^ provides details of aggregate preparation by wet sieving. Briefly, 2–4 mm soil aggregates were wet sieved for 15 minutes by a Yoder apparatus with 15 strokes per minute and 20 mm oscillation depth. The sieves used were 2000, 1000, 500, 250, 125 and 53 μm. The aggregates retained on each sieve were dried to constant moisture content at 40 °C. The 250–2000 μm and 53–250 μm aggregates were referred to as macro- and micro-aggregates, respectively. Ten 250–2000 μm macro-aggregates and ten 53–250 μm micro-aggregates from each land use were randomly selected for μCT analysis. For microbiological analysis a subsample of oven dried (40 °C) macro- and micro-aggregates were kept below 4 °C.

### Image acquisition

Soil aggregates were scanned by a v|tome|xs 240 dual gun X-ray computed tomography unit (Phoenix|X-ray, GE Sensing & Inspection, Wunstorf, Germany) located at the University of New England (Armidale, Australia). Scanning of the aggregates was undertaken using the nano-focus X-ray tube set to focal Mode 1^*®*^ with energy settings of 100 kV and 135 mA. The resolutions of scans for macro- and micro-aggregates were 5.2 μm and 4.0 μm, which were the maximum possible resolutions for both aggregate size classes within the X-ray system. We acquired 720 two dimensional 16 bit TIFF images by 360° rotation of the sample with incremental angle of 0.5° resulting in 512 × 512 × 512 pixels for each image.

### Image reconstruction and analysis

The three dimensional volume was digitally reconstructed from the X-ray images using Datos|x–reconstruction software (Phoenix|X-ray, GE Sensing & Inspection, Wunstorf, Germany). Geometric calibration and beam hardening corrections were undertaken before reconstruction. The reconstructed volumes were analyzed with public domain software Fiji ( http://pacific.mpi-cbg.de/wiki/index.php/Fiji) and SCAMP, the soil specific plugin^39^. The three dimensional pore networks were visualized by VGStudio MAX, Version 2.0 (Volume graphics GMBH, Germany).

For the separation of pores from the soil matrix, image stacks were first cropped to remove image volumes external to the aggregate itself, and also to remove any beam hardening artefacts^39^. The aggregate image stack (8 bit greyscale) was then segmented into pores and solid material using the thresholding tool in Fiji. The initial threshold value was chosen from the intersection region of the bimodal gray level histogram of the image and then adjusted by visual comparison of original and thresholded images^40^. The thresholded images were then converted into a binary image stack showing pores in white and solids in black. These images were used to calculate porosity, pore size distribution, and spectral dimension (i.e. a mathematical expression of pore connectivity and tortuosity^41^) of the macro- and micro-aggregates using SCAMP.

### Size and density fractionation and carbon decomposition rate in soil aggregates

Macro- and micro-aggregates were fractionated into SOC size and density fractions as described by Rabbi *et al*.^39^. Data of occluded particulate organic carbon (oPOC) is presented in this current work. The decomposition rate constant of the slow organic carbon pool (Ksoc) in macro- and micro-aggregates were determined using a two-pool model of organic carbon decomposition as described by Rabbi *et al*.^38^ and Collins *et al*.^42^.

### Diversity of bacteria in soil aggregates

Total nucleic acids were extracted from all samples of one sampling site using the PowerSoil DNA isolation kit (MO BIO Laboratories Inc., Carlsbad, CA, USA) following the manufacturer’s instructions. The extracted nucleic acid specific for bacteria was amplified by PCR with primer set 63F:5′-5CAGGCCTAACACATGCAAGTC-3′ and 1389R:5′-5ACGGGCCGGTGTGTACAAG-3′.The 63F (forward labelled) and 1389R (reverse labelled) primers were labelled with Phosphoramidite dyes FAM and HEX, respectively. The amplified DNA was purified using a QLAquick PCR purification kit (QIAGEN Pty Ltd., Clifton Hill, VIC, Australia) according to the manufacturer’s instruction. The purified PCR product was digested separately with, MspI restriction enzyme. After digestion, TRFLP (Terminal Restriction Fragment Length Polymorphism) analysis was carried out with an AB3730 DNA Analyzer using Liz 1200 internal size standard (Applied Biosystems by Life Technologies, Thermo Fisher Scientific Australia Pty. Ltd., Scoresby, VIC). As the fluorescence intensity of each peak of TRFLP is proportional to the amount of DNA from each operational taxonomic unit of the bacteria in the sample^43^, the diversity of the bacterial community was calculated using the Shannon index^44^,^45^.


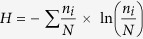


where n_i_ is the height of a peak and N is the sum of all peaks in each sample. Since reverse label TRFs gave better discrimination between samples, forward labelled TRFs were not used for statistical analysis.

### Statistical Analysis

Data were analysed using R version 3.0.2 (R Development Core Team, 2013). Analysis of variance (ANOVA) with a mixed effect model was performed on pore geometry, oPOC and Ksoc, considering land use and aggregate size class as fixed factors and site as a random factor. Data were transformed as needed to meet the assumptions of normality and homogeneity of variances. The diagnostics of the mixed effect model were checked for homogeneity, normality and actual versus fitted values. Since the interaction between aggregate size classes and land uses was not statistically significant, Tukey contrasts were undertaken using MULTCOMP package in R for multiple pair wise comparisons of fixed factors.

A general linear model was used to test the effect of aggregate size classes and land uses on bacterial diversity data. Tukey honest significant difference (HSD) test was performed in R for mean comparison of aggregate size classes and land uses. In addition to the above, differences in the percentage of the 20–40 μm and <20 μm pores between macro and micro-aggregates were determined using a paired t-test. Structural equation modelling was undertaken to determine the relative strength of the relationship of bacterial diversity in aggregates with oPOC, porosity, pore connectivity and Ksoc using using AMOS 21 (IBM SPSS, Amos Development Corporation, Meadville, Pennsylvania, USA). A combination of non-significant chi-square (χ^2^) test, goodness of fit index (GFI) and root mean square error of approximation (RMSEA) were used to find an acceptable structural equation model^46^,^47^. Additionally, we confirmed the fit of the model using the Bollen–Stine bootstrap test^48^. The structural equation model attained an acceptable fit by all criteria. Additional analysis was also performed to include biochemical quality of oPOC^49^, pore classes and land uses in SEM (Supplementary Material S2 and S3). But the SEM did not attain an acceptable fit by all model evaluation criteria.

## Additional Information

**How to cite this article**: Rabbi, S. M. F. *et al*. Physical soil architectural traits are functionally linked to carbon decomposition and bacterial diversity. *Sci. Rep.* **6**, 33012; doi: 10.1038/srep33012 (2016).

## Supplementary Material

Supplementary Information
