# Supplementary material for: Physical soil architectural traits are functionally linked to carbon decomposition and bacterial diversity
Source: Sci Rep. 2016 Sep 12;6:33012. doi: 10.1038/srep33012 (PMC5018812; doi:10.1038/srep33012)
Supplement: Supplementary Information [file srep33012-s1.pdf]

Supplementary Material

Physical soil architectural traits are functionally linked to carbon decomposition and bacterial diversity.

S.M.F. Rabbi[1,2*], H. Daniel[2], P.V. Lockwood[2], C. Macdonald[3], L. Pereg[4], M. Tighe[2], B.R. Wilson[2], I.M. Young[1]

[1]School of Life and Environmental Sciences, University of Sydney, Sydney, NSW, Australia

[2]Plant, Soil and Environment Systems, School of Environmental and Rural Science, University of New England, Armidale, NSW, Australia

[3]Hawkesbury Institute for the Environment, Western Sydney University, Sydney, Australia

[4] School of Science and Technology, University of New England, Armidale, NSW, Australia

** Corresponding author; sfazler2@une.edu.au*

Supplementary material S1 | Description of sampling sites, soil management history, percent sand, silt and clay, bulk density, soil pH, organic carbon concentration in aggregates and clay mineralogy of Dermosols studied under native pasture, crop/pasture rotation and woodland in Northern Tablelands, NSW, Australia (modified from Rabbi et al.[38]).

Sites	Land uses	Geo-reference	History	Sand	Silt	Clay	Bulk density	pH_w (1:5)	Soil organic carbon (SOC)		Dominant Clay Minerals
									250-2000 µm	53-250 µm	
				%			g cm^{-3}		g SOC kg^{-1} aggregate		
Kirby	Native pasture	30°26'S 151°38'E	Native grass, lightly grazed, >20 years under current management	37.49	15.42	47.09	1.01	5.84	43.87	40.67	Smectite>Kaolinite>Illite
	Crop/pasture rotation	30°26'S 151°38'E	Crop/pasture rotation, sporadic fertilizer application and > 20 years under current management	34.04	11.37	54.59	0.92	6.09	45.30	29.57	
	Woodland	30°26'S 151°38'E	Eucalypt woodland, grass cover lightly grazed, >20 years under current management	45.27	15.69	39.04	0.84	6.04	53.20	39.97	
Clarkes	Native pasture	30°28'S 151°38'E	Native grass, lightly grazed, >20 years under current management	46.89	25.38	27.73	0.82	5.86	52.13	46.23	
	Crop/pasture rotation	30°28'S 151°38'E	Crop/pasture rotation, regular fertilizer application and > 20 years under current management	37.92	10.13	51.95	0.72	5.76	44.37	37.70	
	Woodland	30°28'S 151°38'E	Eucalypt woodland, grass cover lightly grazed, >20 years under current management	36.36	16.70	46.94	0.87	6.04	45.60	35.20	
Powalgarh	Native pasture	30°09'S 151°36'E	Native grass, lightly grazed, >20 years under current management	59.58	28.51	11.91	1.09	5.94	24.93	30.13	
	Crop/pasture rotation	30°09'S 151°36'E	Crop/pasture rotation, regular fertilizer application and > 10 years under current management	49.55	28.32	22.13	1.22	5.72	29.63	40.20	
	Woodland	30°09'S 151°36'E	Eucalypt woodland, grass cover lightly grazed, >20 years under current management	62.58	18.99	18.43	0.90	5.83	26.63	38.20	
Black Mountain	Native pasture	30°18'S 151°39'E	Native grass, lightly grazed, >20 years under current management	68.19	10.87	20.94	0.95	5.93	32.97	52.87	
	Crop/pasture rotation	30°18'S 151°39'E	Currently sown to millet, fertilizer application, >10 years under current management	55.56	21.99	22.45	1.13	5.84	30.50	33.80	
	Woodland	30°19'S 151°39'E	Eucalypt woodland, >20 years under current management	51.13	33.61	15.26	0.58	5.91	55.30	51.73	

Supplementary material S2| Relative contents (% of total signal acquired) of alkyl C, O-alkyl C, aromatic C, carboxyl C and alkyl/O-alkyl C ratios of oPOC as revealed by ^{13}C CPMAS NMR spectroscopy (modified from Rabbi et al.[49])

Land use	Chemical Shift limits (ppm)				Alkyl C/ O-Alkyl C
	220-160 Carboxyl C	160-110 Aromatic C	110-45 O-Alkyl C	45-(-10) Alkyl C	
250-2000 µm					
Native Pasture	9.2	18.73	54.98	17.09	0.31
Crop/pasture rotation	9.58	14.66	52.54	23.22	0.44
Woodland	10.64	19.51	47.21	22.63	0.48
53-250 µm					
Native Pasture	12.47	17.11	48.55	21.88	0.45
Crop/pasture rotation	11.59	18.01	48.34	22.06	0.46
Woodland	13.4	23.59	40.09	22.92	0.57

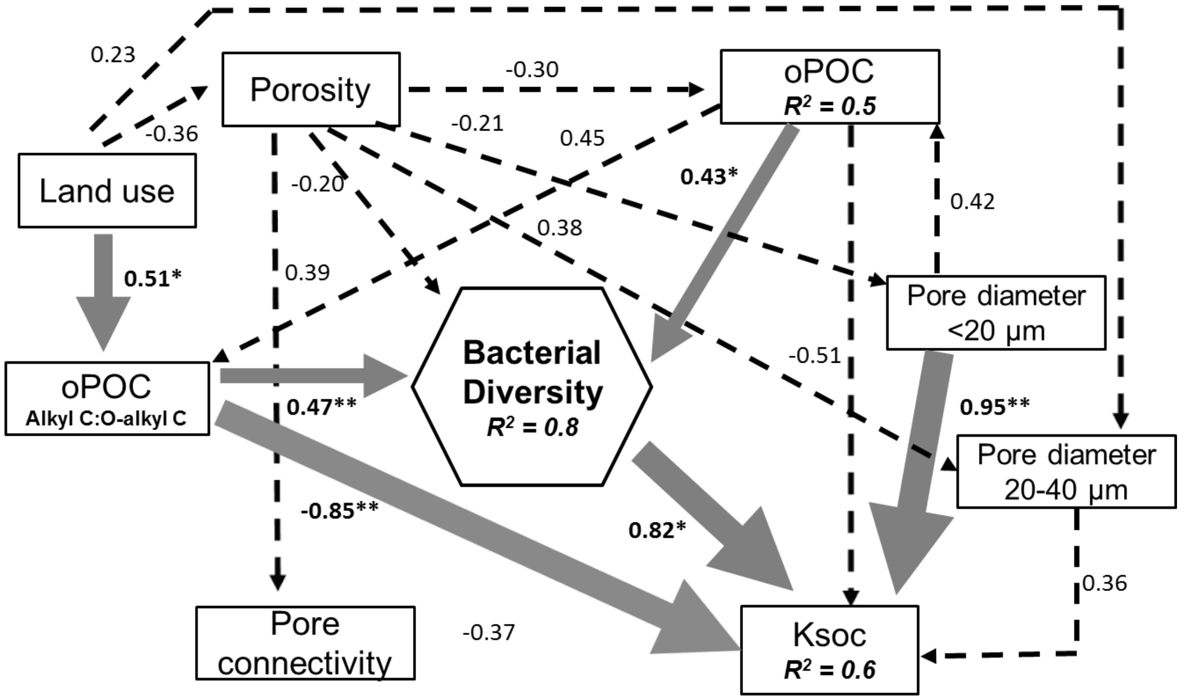

Supplementary material S3 | Relationship of pore geometry with carbon decomposition rate occluded POC (oPOC) and bacterial diversity. The model attained an acceptable fit ($\chi 2 = 6.32$, p = 0.1, df = 3, Bootstrap p = 0.428, RMSEA = 0.29 p = 0.1, AIC = 108.3). The numbers adjacent to the arrows represent standardized path coefficients, analogous to regression weights. The width of each arrow is indicative of effect size. Continuous arrows indicate significant ($p<0.05$ (*) & $p<0.01$ (**)) positive or negative relationships, whereas dashed arrows indicate non-significant relationships ($p>0.05$). The proportion of variance of bacterial diversity, Ksoc and oPOC explained (R^2) is shown in the box for bacterial diversity. The relationships that have standardized path coefficients ≥0.2 are shown in the path diagram.
